# Connecting past and present: single-cell lineage tracing

**DOI:** 10.1007/s13238-022-00913-7

**Published:** 2022-04-19

**Authors:** Cheng Chen, Yuanxin Liao, Guangdun Peng

**Affiliations:** 1 Center for Cell Lineage and Development, CAS Key Laboratory of Regenerative Biology, Guangdong Provincial Key Laboratory of Stem Cell and Regenerative Medicine, GIBH-HKU Guangdong-Hong Kong Stem Cell and Regenerative Medicine Research Centre, Guangzhou Institutes of Biomedicine and Health, Chinese Academy of Sciences, Guangzhou, 510530 China; 2 Center for Cell Lineage and Atlas, Bioland Laboratory (Guangzhou Regenerative Medicine and Health Guangdong Laboratory), Guangzhou, 510005 China; 3 Institute for Stem Cell and Regeneration, Chinese Academy of Sciences, Beijing, 100101 China; 4 University of Chinese Academy of Sciences, Beijing, 100049 China

**Keywords:** single-cell lineage tracing, integration barcodes, polylox barcodes, CRISPR barcodes

## Abstract

Central to the core principle of cell theory, depicting cells’ history, state and fate is a fundamental goal in modern biology. By leveraging clonal analysis and single-cell RNA-seq technologies, single-cell lineage tracing provides new opportunities to interrogate both cell states and lineage histories. During the past few years, many strategies to achieve lineage tracing at single-cell resolution have been developed, and three of them (integration barcodes, polylox barcodes, and CRISPR barcodes) are noteworthy as they are amenable in experimentally tractable systems. Although the above strategies have been demonstrated in animal development and stem cell research, much care and effort are still required to implement these methods. Here we review the development of single-cell lineage tracing, major characteristics of the cell barcoding strategies, applications, as well as technical considerations and limitations, providing a guide to choose or improve the single-cell barcoding lineage tracing.

## Introduction

Cells are the basic units of all living organisms. As a long-standing goal, depicting a cell’s origin, current state, and future fate in physiological and pathological contexts presents a challenging task for biomedical research. Recent advances in single-cell transcriptomics hold great promises to elucidate the molecular mechanisms of cell fate determination of animal development and stem cell differentiation (Hwang et al., 2018; Lafzi et al., 2018; Potter, 2018; Svensson et al., 2018). By simultaneously measuring the expression of multiple genes in individual cells, single-cell RNA sequencing (scRNA-seq) greatly facilitates the characterization of cell states with a large dimensionality. Additionally, based on pseudotemporal ordering (sequence of gene expression change) or mRNA splicing dynamics, computational tools, such as Monocle and RNA velocity, have been conceived to infer cell state trajectories, offering an attainable approach to determine molecular dynamics of thousands of genes (La Manno et al., 2018; Saelens et al., 2019). Armed with the analytic power of scRNA-seq, many investigations attempt to provide a comprehensive molecular cell atlas of animal development at the single-cell level, which are accompanied with identifying new cell types and illustrating the major cell lineage segregation, as well as characterizing molecular markers or drivers (Briggs et al., 2018; Farrell et al., 2018; Zhong et al., 2018; Cao et al., 2019; Packer et al., 2019; Pijuan-Sala et al., 2019; Tam and Ho, 2020; Mittnenzweig et al., 2021). Besides, numerous studies have been carried out to delineate the cell fate transitions in both 2D stem cell culture and 3D organoids (Treutlein et al., 2016; Camp and Treutlein, 2017; Semrau et al., 2017; Han et al., 2018). Similarly, scRNA-seq data continue to grow rapidly with the progress of cell atlas projects (Regev et al., 2017; Bock et al., 2021).

As mentioned earlier, current research based on single-cell transcriptomics heavily relies on pseudotemporal ordering to infer cell trajectories and connective lineage relationships. However, route descriptions of state transitions may or may not be equivalent to the true lineage paths of a progenitor population, and severe limitations remain to be resolved before accurate inferences can be achieved. First, a confident inference of cell trajectory requires substantial sampling of cells, which may be impeded by the incomplete or insufficient coverage of scRNA-seq in some cases (Tanay and Regev, 2017). Second, cell fates may converge in specific situations. A well-known example of lineage convergence is that the definitive endoderm has both extra-embryonic and embryonic origins (Kwon et al., 2008; Chan et al., 2019). Third, the cell state transition may undergo rapid divergence with the change of a few molecular drivers, which is challenging to distinguish with transcriptome analysis alone (Wagner et al., 2018; Packer et al., 2019; Tritschler et al., 2019; Wagner and Klein, 2020). Alternatively, by utilizing the dynamics of unspliced and spliced mRNAs, RNA velocity adds a new layer of information (molecular kinetics) to clarify the direction of cell state transitions. However, the ability to infer cell fate transitions for RNA velocity relies on assumptions such as constant gene-specific splicing rates, which may be violated in some situations (La Manno et al., 2018; Svensson and Pachter, 2018; Tritschler et al., 2019; Bergen et al., 2020; Lederer and La Manno, 2020). Altogether, although current computation tools may reveal the cell state trajectories by various pseudo-time analysis algorithms, more defined information with the ability to record the cell history is crucial to fully clarify the true path of lineage specification.

As a substantial improvement in depicting and predicting cell behaviors, recent developments of single-cell lineage tracing, which combines single-cell omics and lineage tracing, enable detecting cell state and clonal relationship in parallel, thereby illustrating a credible route of lineage segregation by integrating the information of the clonal history and state transition. As a cutting-edge technique, extensive reviews have discussed various aspects of its basic concepts, sequence architectures, computational tools, and so on (Woodworth et al., 2017; Kebschull and Zador, 2018; Kester and van Oudenaarden, 2018; Baron and van Oudenaarden, 2019; McKenna and Gagnon, 2019; Wu et al., 2019; VanHorn and Morris, 2020; Wagner and Klein, 2020). In this technical review, we first attempt to describe the whole path of technical advances leading to the single-cell resolution of lineage tracing. We then focus on current implementations for three barcoding strategies widely adapted in experiments. Furthermore, we summarize several scenarios to apply single-cell lineage tracing. Finally, we discuss some technical considerations and caveats of current barcoding strategies.

## From traditional lineage tracing to single-cell lineage tracing

Lineage tracing is the gold standard to infer the relationship between progenitors and their progenies. By labeling the progenitors and examining their locations and marker expressions at a later time point, the lineage segregation that occurred during this interval can be queried. Further lineage structure could also be constructed by consecutively labeling the progenitors at different developmental stages (Buckingham and Meilhac, 2011; Blanpain and Simons, 2013; Hsu, 2015; Wagner and Klein, 2020). Lineage tracing strategies can be roughly divided into two categories: prospective tracing and retrospective tracing. Prospective tracing approaches label the progenitor cells and identify their descendants with the same tag. In contrast, retrospective tracing approaches access the natural genetic markers accumulated in the progenies across multiple cell divisions and then infer their lineage relationship through shared markers (Girskis and Woodworth, 2016; Woodworth et al., 2017; Baron and van Oudenaarden, 2019; Figueres-Onate et al., 2020; Wagner and Klein, 2020).

**Figure. 1:**
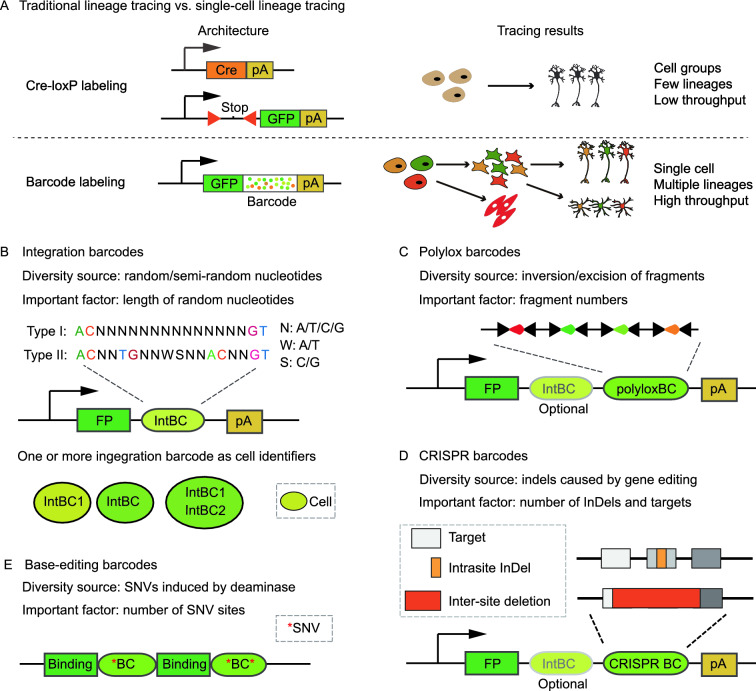
Barcoded-based single-cell lineage tracing. (A) Comparison between Cre-LoxP labeling and DNA barcode labeling. (B) Integration barcodes. One or multiple integration barcodes can be used as cell identifiers to record clonal information. (C) Polylox barcodes. (D) CRISPR barcodes. In most cases, most InDels occur within a single target (intrasite InDel), but a deletion spanning multiple targets may happen when Cas9 cuts two targets at the same time (inter-site deletion). As illustrated, a middle intrasite InDel may be eliminated by inter-site deletion. IntBC, integration barcode; polyloxBC, polylox barcode; CRISPR BC, CRISPR barcode; FP, fluorescent protein; pA, polyA tail. (E) Base-editing barcodes. SNVs induced by base editors are usually adjacent to the DNA binding motifs

Tracking all the offspring of a single cell to define its cell lineage (i.e., prospective tracing) has a long history dating back to the early ages of developmental biology. The initial implementation of lineage tracing relies on direct observation and manual labeling using vital dyes. Although it’s possible to reach single-cell resolution, these techniques are labor-intensive and usually limited to transparent samples. In the era of modern molecular genetics, fluorescent proteins, whose expressions are usually under the control of site-specific recombinases such as Cre, become the conventional “molecular dyes” to track cells. However, as the expressions of site-specific recombinase are usually controlled by cell-type-specific promoters, which in turn confine the expressions of fluorescent proteins in a group of cells instead of a single cell. To increase the resolution of lineage tracing, multicolor labeling systems such as Brainbow and Confetti are developed. By putting multiple flox sites and/or multiple fluorescent proteins in specific combinations, Brainbow or Confetti may generate dozens to hundreds of colors to distinguish different cells upon Cre activation. Nevertheless, multicolor labeling remains challenging to reach single-cell resolution due to the complicated trials of time and dose on initiating labelling (Buckingham and Meilhac, 2011; Hsu, 2015; Weissman and Pan, 2015; Liu et al., 2020). In contrast, DNA barcodes are DNA fragments with huge sequence variations and their advent offers a novel approach to label individual cells (Fig. 1A). Retroviral libraries of DNA barcodes allow for labeling thousands of hematopoietic stem cells (HSCs) and tracking their cell fates simultaneously, which greatly enhance the tracing contents and resolution (Gerrits et al., 2010; Lu et al., 2011; Naik et al., 2014; Weinreb et al., 2020). However, viral barcoding is not easy to be precisely implemented in some *in vivo* settings. To address this problem, new genetic labels such as polylox barcodes and CRISPR barcodes have been developed. Their versatile applications are capable of labeling a single progenitor *in vivo*, for some barcodes are generated in low probabilities, making it unlikely to contain the same barcode among multiple cells (McKenna et al., 2016; Pei et al., 2017; Kalhor et al., 2018). Remarkably, like somatic mutations, cumulative CRISPR/Cas9 insertions and deletions (InDels) can also serve as genetic landmarks to reconstruct the lineage hierarchy of different cells (Baron and van Oudenaarden, 2019; Espinosa-Medina et al., 2019; Spencer Chapman et al., 2021). A more recent breakthrough for lineage tracing is the development based on base editors, which offers more informative sites to record the cell division events (Liu et al., 2021). Of note, compared to somatic mutations, InDels and mutations induced by CRISPR-based genome editing can record more mitotic divisions due to their faster mutation rates, thus generating a more elaborate cell lineage tree. In summary, the trend of lineage tracing is towards finer (from distinguishing a group of cells to a specific cell) and broader (from tracing a few cells to thousands of cells) resolutions of both progenitors and their offspring.

Nevertheless, detecting thousands and millions of barcodes that label thousands of progenies is a strenuous task. In this regard, transcribing the barcode information into the mRNA pool and retrieving these barcodes by high-throughput sequencing such as scRNA-seq is more practical. Importantly, scRNA-seq at the end of lineage tracing also assists the characterization of progenies’ phenotypes (Raj et al., 2018; Wagner et al., 2018; Pei et al., 2020; Wagner and Klein, 2020). This combined effort to distinguish thousands of clones and a variety of cell types leads to the birth of single-cell lineage tracing (SCLT). In brief, compared to classical prospective or retrospective lineage tracing, SCLT shows two paramount features. First, SCLT can identify the progenitor-progeny relationship by barcode evolution and define the states of each cell by scRNA-seq, thus enabling the revelation of integrated lineage kinship and molecular trajectory. Second, thousands of clones can be analyzed in parallel to refine the lineage connections, representing an unbiased and large-scale survey of cell-to-cell transitions.

## Barcode classification

The genetic markers for SCLT can be roughly divided into two categories: endogenous markers and exogenous markers. Common endogenous markers include nuclear genome changes (e.g., SNP, CNV, microsatellites repeat, L1 retrotransposition elements) and mitochondrial genome changes. These endogenous markers usually arise from the random errors of DNA replication, DNA repair, or random transposon integration in chromosomes. It’s feasible to reconstruct the complete or partial cell lineage by deducing the pattern of mutation heredity. However, the readout of somatic mutations usually requires costly deep sequencing of the whole genome or exome of single cells, given the low frequency of these somatic mutations and their sparse genomic distribution (Woodworth et al., 2017; Baron and van Oudenaarden, 2019; Bizzotto et al., 2021; Spencer Chapman et al., 2021). Nevertheless, a few studies take advantage of mutations in the mitochondrial DNA for their high mutation rate, high copy number, and high levels of heteroplasmy. This strategy reduces the cost of sequencing but may suffer from the horizontal transfer of mitochondria between cells (Ludwig et al., 2019; Lareau et al., 2021). Recently, some researchers tried to use CRISPR/Cas9 or base editor to target some specific endogenous loci of the genome. These modifications increase the frequency of detectable mutations, but might violate the normal development of cells (Hwang et al., 2019; Cotterell et al., 2020; Ye et al., 2020).

To avoid the limitations of endogenous markers, three major types of exogenous markers (integration barcode, polylox barcode, CRISPR barcode) have been established for experimental systems that are compatible with genetic manipulations (Fig. 1B–D). These exogenous markers are usually packed in a defined region and transcribed by constitutive promoters to facilitate their detection by high-throughput sequencing methods such as scRNA-seq and amplicon sequencing. As these exogenous markers can achieve high resolution of lineage tracing in the experimentally tractable system, comprehensive descriptions of these strategies are discussed here. Besides, a brief introduction on base-editing barcodes, which are considered as a promising next-generation lineage tracer, is arranged after the descriptions of CRISPR barcodes due to their inherent similarity (Jones et al., 2020). However, unless in related sections, they will not be further discussed as they await future studies to witness their full potential in lineage tracing.

### Integration barcodes

Integration barcodes are originally introduced as short stretches of DNA sequences that flank the integration sites of transposons or lentiviruses. As the transposons or lentiviruses randomly integrate into genomes, the flanked sequences are also random, thus serving as unique clone tags to distinguish cells with different integration events. Although the number of such integration barcodes seems unlimited, it’s hard to interrogate their information with scRNA-seq. To address this issue, the current version of integration barcodes is designed as short DNA fragments placed in an expressed locus, and they can be synthesized as type I barcodes (consecutive random nucleotides) or type II barcodes (random nucleotides interspersed with fixed nucleotides) (Lu et al., 2011; Rodriguez-Fraticelli et al., 2018). Integration barcodes are commonly used as barcode libraries, and the library diversity is determined by the total length of random nucleotides (Fig. 1B). Theoretically, the diversity of a barcode library with 10 bp random nucleotides can approach 1 million, although such diversity is hard to achieve after multiple steps of molecular cloning. Generally, type II barcodes can avoid consecutive poly-N regions, which are unfriendly for oligo synthesis, PCR amplification, and further sequencing, hence reducing the loss of barcode diversity at the cost of longer sequencing length. Besides, semi-random sequences (“N” is replaced by “S/W”) have also been applied for barcode design. This design maintains a constant GC ratio while sacrificing the barcode diversity (Bystrykh and Belderbos, 2016; Bramlett et al., 2020).

Integration barcodes are typically inserted in the 3′UTR after the coding region of fluorescent proteins. This architecture makes it convenient to retrieve the barcoded cells by fluorescence-activated cell sorting (FACS). A common application of integration barcodes is to label thousands of cells (e.g., HSCs and cancer cells) at the onset of lineage tracing and then evaluate their clonal dynamics. For this purpose, lentiviruses are preferred due to their high degree of transduction efficiency (Gerrits et al., 2010; Lu et al., 2011; Bystrykh et al., 2014). Other transgene systems have also been employed. For example, Tol2 transgenesis can introduce integration barcodes to the zebrafish genome in an accumulative way during early embryogenesis (Wagner et al., 2018).

### Polylox barcodes

As mentioned earlier, integration barcodes are not easily adapted for *in vivo* systems, and one alternative is polylox barcodes. The intact form of polylox barcodes is a DNA cassette with multiple loxP sites in alternating orientations (Fig. 1C). Under the activity of Cre, the intervened DNA fragment between two loxP sites will be excised if these two sites are in the same orientation, whereas inverted if these two sites are in the opposite orientation. In polylox, recombination events can happen between any two loxP sites, and recombination won’t stop until usable loxP sites are exhausted or the Cre activity stops. The fast recombination of multiple loxP sites could generate thousands of recombination barcodes in a relatively short interval, thus labeling thousands of cells at the onset of lineage tracing with extremely high barcode diversity. In the first implementation, an intact polylox fragment with 10 loxP sites is introduced into the *Rosa26* locus by targeted insertion and interrogated by amplicon sequencing, and the single-cell resolution is accomplished by FACS of the interested cells into 8-tube strips individually. Strikingly, an estimated maximum of 1.8 million distinct barcodes can be generated by random recombination of the polylox fragment (Pei et al., 2017, 2019). A later version of this method named polyloxExpress offers a more friendly approach to query the cell types by placing the polylox fragment in the 3′UTR of tdTomato fluorescence reporter, whose expression is driven by *Rosa26* promoter (Pei et al., 2020). Both implementations have been tested in mice to track the clonal behavior of HSCs. The polylox design takes advantage of the abundant Cre mouse lines that have been generated during the past few decades. With a simple cross between polylox mice and tissue-specific Cre mouse line, this method achieves clonal tracking in the selected tissues. Currently, one major concern for polylox barcodes is the requirement of expensive long-read sequencing. A few conceptual designs of polylox barcodes for short-read sequencing have been proposed, but their feasibility in mammalian cells remains elusive (Peikon et al., 2014; Weber et al., 2016).

As the length of integration barcodes is short, it is intuitive to combine integration barcodes with polylox fragments. In this situation, integration barcodes function as identifiers to distinguish the different copies of polylox fragments introduced by random insertion (Fig. 1B). Using random sequences as integration identifiers greatly increases the barcode diversity. For example, without considering integration identifiers, only 155 barcodes are generated by polylox recombination, while 514 barcodes are retrieved when distinguishing the same recombination by integration identifiers (Kim et al., 2020).

### CRISPR barcodes

The CRISPR barcodes are combinations of InDels generated by CRISPR/Cas9 mediated genome editing (Fig. 1D). The proof of concept using CRISPR barcodes for lineage tracing *in vivo* was firstly demonstrated in zebrafish by targeting a tandem array of Cas9 targets, which is integrated into the genome by Tol2 transgenesis (McKenna et al., 2016). This success was followed by a bunch of different designs, and the shared principle of these designs can be described as follows: multiple targets are introduced into genomes either at one locus or multiple loci. As the editing rate of Cas9 nuclease is limited, the edited targets are accumulated after multiple rounds of cell divisions until saturated. Generally, dozens to hundreds of InDels can be generated in one target. With the assumption that targets are independent of each other, thousands of barcodes (InDel combinations across multiple targets) are accumulated in different cells. By accessing the barcode information at the end of lineage tracing, it’s possible to reconstruct cell lineage trees based on shared InDels.

To date, there is no common practice about how to implement CRISPR barcodes. Here the current practices of CRISPR barcodes are categorized into three types. The first type, as reported in ScarTrace or LINNAEUS designs, selects the Cas9 targets in the GFP or RFP coding regions so that InDels are indicated by the attenuation of the fluorescence intensity (Alemany et al., 2018; Spanjaard et al., 2018). Although ScarTrace and LINNAEUS designs take advantage of available transgenic zebrafish lines, the calling of barcodes may be suffered as it’s difficult to distinguish the same type of InDels from different loci, thus undermining their abilities to reconstruct accurate lineage trees. Instead of having one Cas9 target at one integration locus, the second type of CRISPR barcodes, such as scGESTALT and CARLIN designs, usually places multiple Cas9 targets together to form a tandem array in the 3′UTR of GFP/RFP. These designs greatly facilitate the simultaneous detection of multiple targets (Raj et al., 2018; Bowling et al., 2020). The third type of CRISPR barcodes is generated from MARC1 design. In this design, the gRNAs are called homing gRNAs, since the gRNA scaffolds are modified so that the expressed gRNAs can target their own loci for several rounds until the length of guide sequences is out of range. As the InDels are obtained by several rounds of self-targeting, this type of barcodes generally achieves higher diversity per target (Kalhor et al., 2018). Notably, the current practice of MARC1 hasn’t been adapted with scRNA-seq yet, as the capture of homing gRNAs using scRNA-seq awaits further modification.

With regards to the genomic location of targets, there are distributed arrays and tandem arrays. Tandem arrays are implemented by targeted insertion to genomic safe harbors such as *Col1a1* locus, while distributed arrays are introduced by random insertions. Similar to polylox barcodes, random insertion can also take advantage of integration barcodes to discriminate different integration copies. Remarkably, multiple integration barcodes in a cell can be viewed as clone identifiers, which help distinguish different progenitors in a progenitor pool (Fig. 1B) (Quinn et al., 2021). Finally, the expression of Cas9 also varies from experiment to experiment. Cas9 expression can be accomplished by direct injection of Cas9 protein/mRNA in zebrafish or by breeding with Cas9-expressing mouse lines (McKenna et al., 2016; Chan et al., 2019; Bowling et al., 2020). However, the best practice on CRISPR barcodes awaits further improvements on various aspects of experimental considerations such as barcode design, barcoding induction timing, tree reconstruction algorithms.

Similar to CRISPR barcodes, base-editing barcodes are combinations of single nucleotide variations (SNVs) generated by base editors. Base editors are initially designed for precise gene editing based on dCas9 (an inactive form of Cas9) before their adaption for lineage tracing. They are special nucleases composed of three domains, a DNA-binding domain (e.g., dCas9) which allows the base editors to search for DNA targets, a deaminase domain that transforms the adjacent nucleotides (e.g., C to U mutations caused by activation-induced cytidine deaminase), and a UGI (uracil DNA glycosylase inhibitor) domain which inhibits associated repair systems that may suppress the transformations. Compared to CRISPR barcodes, a unique feature of base editors is their ability to record cell division events, since DNA replication is essential for the successful transformation of nucleotides induced by deaminases (Rees and Liu, 2018). As the scanning space of deaminases is limited by DNA binding domains, only a few nucleotides adjacent to the DNA binding sites will be edited. To increase the number of editable sites, multiple DNA binding sites are preferred architectures for base-editing barcodes (Fig. 1E). Using a dCas9-derived base editor, Hwang et al. proved the concept of tracing cell lineages by targeting the endogenous L1 retrotransposition elements (Hwang et al., 2019). Besides dCas9, other DNA binding proteins such as iSceI could also be applied in base editors in the setting of lineage tracing. One exemplary demonstration of the usage of iSceI has been suggested by a recently published design called SMALT. This work took advantage of base editing on an exogenous synthetic three-kilobase DNA cassette containing many editable sites, and provided many informative sites for recording thousands of cell division events in fruit flies (Liu et al., 2021). Considering the high recording ability of base-editing barcodes, their future integration with scRNA-seq may be promising for dissecting the lineages on organism level.

## Current and promising applications of SCLT

SCLT can leverage the information from both lineages and single-cell transcriptomes. As a result, SCLT can offer unprecedented perspectives to analyze clonal histories and molecular mechanisms in qualitative and quantitative manners. The following points are some of its fascinating applications.

### Clarifying clonal relationships between different cell types

**Figure. 2:**
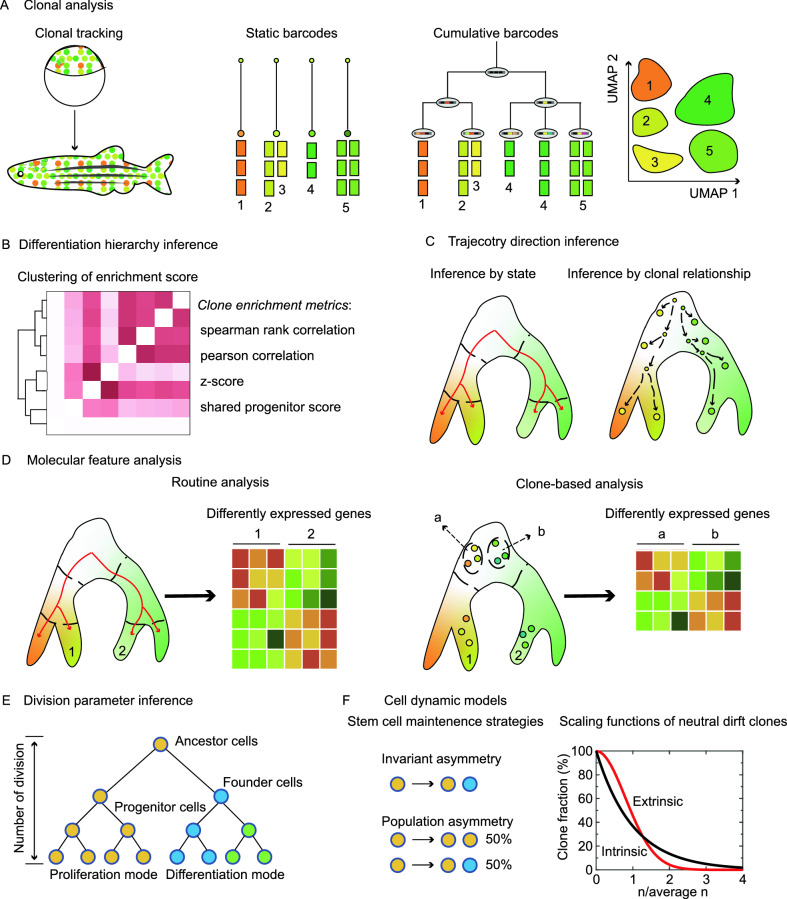
Current and promising applications of single-cell lineage tracing. (A) Two types of barcodes for clonal analysis. In the setting of single-cell lineage tracing, characterization of cell types is usually achieved by dimensionality reduction such as uniform manifold approximation and projection (UMAP). (B) Some clone enrichment metrics for lineage coupling analysis [figure adapted from (Chan et al., 2019)]. (C) Trajectory direction can be determined by integrating state and clonal information. (D) Dividing transcriptionally similar cells into subgroups by clones facilitates the identification of delicate molecular signatures involved in cell fate bias. (E) Inferring basic parameters for a cell division tree such as the number of founder cells, the number of cell divisions, and cell division modes. (F) Building mathematical models to understand the cell behaviors [adapted from (Klein and Simons, 2011)]

Clonal tracing by barcodes makes it possible to identify the proliferation, migration, apoptosis, and differentiation events of individual cells (Petit et al., 2005; Buckingham and Meilhac, 2011; Blanpain and Simons, 2013; Naik et al., 2014). Barcodes can be either static or cumulative depending on whether their sequence identity evolves during developmental processes. If barcodes are invariable once introduced, then the barcodes are static; In contrast, if barcodes keep evolving, then the barcodes are cumulative (Fig. 2A). Static barcodes allow for revealing clonal potency (self-renewing, unipotent, bipotent, or multipotent) directly by identifying the cell types sharing the same barcode. Generally, the labeled progenitors that are located at the upper level of the lineage hierarchy should have more progenies than those at the lower level. If the progenies derived from earlier barcoded cells are still progenitors, the earlier barcoded cells should have undergone self-renewal. If the offspring is categorized into two differentiated cell types, the labeled ancestors should be considered as bipotent cells. The same logic can also be adapted to identify unipotent and multipotent progenitors. In comparison, cumulative barcodes can record the history of mitotic divisions, so a clonal tree with multi-layers could be reconstructed, then the subclonal relationships between different cell types can be uncovered by analyzing the clonal trees from root to tip, although the granularity varies greatly in different experiments.

Different types of barcodes can serve as either static barcodes or cumulative barcodes depending on their actual usages. Integration barcodes are usually used to label a pool of progenitors as static barcodes, which is a common technique to interrogate the potency of HSCs (Lu et al., 2011; Weinreb et al., 2020). However, integration barcodes can also be applied cumulatively by multiple rounds of integration across a developmental process, as demonstrated in the zebrafish embryogenesis and somatic reprogramming (Biddy et al., 2018; Wagner et al., 2018). As for polylox barcodes, they are primarily generated within a short time interval due to the rapid reaction of LoxP sites once attacked by Cre proteins, so they are currently deemed as static barcodes to track the cell fates of HSCs and pluripotent stem cells (Kim et al., 2020; Pei et al., 2020). Although the generation of CRISPR/Cas9 InDels is inherently cumulative, CRISPR barcodes may also be considered static in some settings such as HSC regeneration and fin regeneration, as they have stopped altering their sequence identity before the concerned developmental events (Alemany et al., 2018; Bowling et al., 2020).

### Resolving lineage relationships by lineage coupling analysis

Knowledge of cell differentiation hierarchies is important for the understanding of cell fate decisions and controlled differentiation *in vitro*. However, it’s difficult to directly elucidate the lineage hierarchy of different cell types by evaluating the clones that share static barcodes. To decipher the lineage hierarchy, statistical analysis may provide a solution by gathering the information from multiple barcodes. Generally, more features are shared between siblings than cousins. Equivalently, the more barcodes are shared between two cell types, the closer the lineage relationship between these two cell types. By calculating the normalized shared barcodes, it’s feasible to infer the lineage relationships between different cell types by hierarchical clustering. Multiple metrics for normalizing shared barcodes have been developed (Fig. 2B). Some of these metrics are spearman rank correlation, observed/expected ratio, correlation of Z-score enrichment, and normalized covariance (Raj et al., 2018; Pei et al., 2019; Weinreb and Klein, 2020; Weinreb et al., 2020). Furthermore, a unique metric called shared progenitor score is applied to deduce lineage hierarchies encoded in cumulative CRISPR barcodes (Chan et al., 2019). Nevertheless, their ability to fully capture the true lineage relationships may need further validation.

### Analyzing trajectory directions in state-fate maps

To unravel transition directions, trajectory analysis algorithms such as Monocle start creating an unrooted tree, and then trajectory directions are established by selecting the root and leaves according to annotated cell states (Qiu et al., 2017). In many cases, trajectory analysis algorithms have good performances to illustrate the sequential cell transitions. However, as mentioned earlier, trajectory analysis may fail to recapitulate the true differentiation paths with states alone under certain circumstances. The SCLT method can address this issue by accessing the shared barcodes, which connect cells with states in the early and late developmental stages (Fig. 2C). For example, by evaluating the single-cell transcriptomes connected by shared polylox barcodes, Pei et al. validated the two major developmental branches (i.e., lymphoid development and myelo-erythroid development) of the mouse *in vivo* hematopoiesis (Pei et al., 2020). Besides, with the aid of clonal information collected from integration barcodes, systematic single-cell evaluation of the relationship between state and fate has been performed in zebrafish, uncovering some major convergent and divergent events of fate transitions during zebrafish embryogenesis (Wagner et al., 2018). Cumulative integration barcodes with inherent information of lineage layers also help to discover the two paths of fate determination in somatic reprogramming from fibroblasts to endoderm progenitors (Biddy et al., 2018). Likewise, single-cell transcriptomic analysis assisted by CRISPR barcodes inherently contains cell trajectory information if InDels are accumulated across the concerned developmental stages (Fig. 2A) (Chan et al., 2019; Bowling et al., 2020).

### Identifying molecular signatures with the support of clonal information

After obtaining single-cell sequencing data, a common practice of finding molecular signatures is to compare the differential expressed genes between different conditions, such as cell types between different lineages or different developmental stages within the same lineages (Van den Berge et al., 2020). Generally, hundreds of differentially expressed genes can be found, while it’s difficult to pinpoint which genes are crucial for the fate choice as the state difference is already manifest between two conditions in the state maps.

The advent of SCLT offers a new route to determine molecular signatures by incorporating clonal information. With the assumption that there exists little fate divergence between recently divided sister cells in a progenitor pool, it’s possible to divide the progenitor pool into different groups of cells with fate biases by utilizing shared clonal information between progenitors and progenies. By comparing different progenitor groups, it’s more likely to identify the crucial regulators involved in fate decisions within dozens of differentially expressed genes. A similar strategy can be applied to determine molecular markers for those transcriptionally similar cells from other events by separating the cells into different groups according to the clonal relationships (Fig. 2D). For instance, by comparing the HSC populations with low progeny output and high progeny output, Rodriguez-Fraticelli et al. discovered dozens of genes that might be involved in regulating the activity of HSCs, and genetic knockouts further validated that one of the candidate genes participated in the quiescence and long-term self-renewal of HSCs (Rodriguez-Fraticelli et al., 2020; Weinreb et al., 2020). Likewise, Biddy et al. identified a candidate gene named *Mettl7a1* by comparing successfully reprogrammed clones and dead-end clones. In addition, they found that this gene could enhance the reprogramming efficiency when adding to the reprogramming cocktail (Biddy et al., 2018). Furthermore, an *in vivo* investigation on HSCs showed that some of the candidate genes derived from this strategy were engaged in proliferation activity or showed lineage biases (Bowling et al., 2020; Pei et al., 2020).

### Dissecting cell dynamics in division hierarchies

Despite inferring tree structures of cell lineages, the quantitative clonal analysis also allows for reckoning cell dynamics. As symmetric divisions boost cell proliferation and asymmetric divisions lead to cell differentiation, plotting clonal distribution and size can distinguish whether cells in a clone undergo symmetric or asymmetric divisions (Petit et al., 2005). For CRISPR barcodes, basic concepts and practices drawn from population genetics using somatic mutations may provide solutions to estimate some parameters of cell dynamics. For example, by calculating the lower threshold of a self-defined parameter called mosaic fractions (MF) for somatic single nucleotide variants that appear in all germ layers, quantitative clonal analysis has been applied to estimate the effective number of epiblast cells (1/MF) in human embryogenesis (Bizzotto et al., 2021). Interestingly, CRISPR barcodes have been adopted to deduce the number/composition of progenitors if the clonal tree has a sufficient resolution (Chan et al., 2019). Compared to CRISPR barcodes, base-editing barcodes generally have more informative sites to infer the cell division events. In their SMALT design, Liu et al. demonstrated that they might simulate the dynamics of the number of actively dividing parental cells in fruit flies, offering an overview of the organ development of fruit flies (Liu et al., 2021). Remarkably, cumulative somatic mutations could be used to estimate the number of cell divisions (Wasserstrom et al., 2008), this strategy might also be adapted by CRISPR barcodes and base-editing barcodes comprised of cumulative sites (Fig. 2E). However, the ability to truly capture the cell division events should be different between CRISPR barcodes and base-editing barcodes. As mentioned earlier, the successful generation of base-editing barcodes requires DNA replication occurring in cell divisions, and this coupling provides foundations for estimating the number of cell divisions (Rees and Liu, 2018). In contrast, the InDels generated by CRISPR/Cas9 may occur at any stage of the cell division. Nevertheless, the internal nodes in the cell phylogenetic tree reconstructed from CRISPR barcodes may capture the independent gene editing events after the division of ancestral cells, which might still be informative to infer the number of cell divisions.

### Population-level models of cell dynamics

Generating or maintaining a functional organ requires an adequate number and composition of cells. Some quantitative studies have shed light on cell dynamics by mathematical or statistical models at the population level (MacLean et al., 2017). One of the most studied models is the neutral drift of adult stem cell clones. As adult stem cells must proliferate and differentiate into new terminal cells to replenish the old or dead ones, they require self-renewal to maintain the total number of adult stem cells. Instead of self-renewing through invariant asymmetric divisions for all adult stem cells as previously suspected, however, recent studies suggested that they adopted a population strategy. In this model, stem cells are equipotent: some stem cells proliferate and some stem cells undergo differentiation, resulting in the neutral drift of stem cell clones. Furthermore, it’s possible to infer the self-renewing properties from intrinsic or extrinsic signals by determining the scaling functions of clonal distribution (Fig. 2F) (Klein et al., 2010; Klein and Simons, 2011). Apart from adult stem cells, mathematical modeling on retina histogenesis also implied that retina progenitors might also be equipotent and showed stochastic clone size in development, while the overall composition of terminally differentiated cell types remained proportioned (He et al., 2012; Zechner et al., 2020). A more recent example also demonstrated the power to combine quantitative lineage analysis and mathematical modeling. Willnow et al. showed that quantitative measurement of the rudiment size by Cre-loxP labeling found that the size of liver bud increased faster than the pancreato-biliary bud, and this phenomenon was not due to different proliferation activities. Using a mathematical simulation on a cell plasticity model, they suggested the existence of a population of multipotent progenitors that could generate both liver progenitors and pancreato-biliary progenitors, which was confirmed by further lineage tracing experiments (Willnow et al., 2021). Of note, few of the above statistical models or mathematical models on cell fate decisions are built based on SCLT data. As SCLT intrinsically acquires enormous information of clones at the population level, it might be promising to revisit those models in adult stem cell systems or build new models to disentangle developmental processes.

## Technical considerations and limitations

The implementation of SCLT contains several major steps: barcode introduction, barcoding initiation, detection of both transcriptomes and barcodes, and computational analysis. Here we discuss several common issues during these steps.

### Random insertion and targeted insertion

Integration barcodes are usually introduced into genomes by random integration, while polylox recombination elements and Cas9 targets are integrated into genomes either by random insertion or targeted insertion. The choices of random insertion or targeted insertion depend on experimental objectives, as these two methods have their own advantages and drawbacks. Given the wide application of random insertion in mutagenesis or enhancer trap, it’s possible that random integration disrupts the regulatory elements and changes the cell state without killing the cells, which may mislead the interpretation of biological results (Sivasubbu et al., 2007; Trinh le and Fraser, 2013). Besides, the expression of barcodes might be suppressed by surrounding transcription-repressive elements. Furthermore, screening for a stable transgenic mouse line after random insertion takes huge efforts, and may cause potential defects when some integration sites become homogeneous. Targeted insertion into the genomic safe locus can guarantee the gene expression and avoid detrimental developmental defects (Papapetrou and Schambach, 2016), but barcode diversity will decrease due to the compromised copy numbers of polylox recombination elements or Cas9 targets. Additionally, inter-site deletions occur in the tandem arrays of Cas9 targets when Cas9 simultaneously cuts two targets (Fig. 1D) (Kalhor et al., 2018; Chan et al., 2019; Bowling et al., 2020), which reduce the barcode diversity and may even eliminate the previous InDel information recorded in the targets between those two targets (Salvador-Martinez et al., 2019). In contrast, this limitation can be avoided by base-editing barcodes as only point mutations occur in the DNA fragments (Fig. 1E).

### Time points or interval of barcoding

**Figure. 3:**
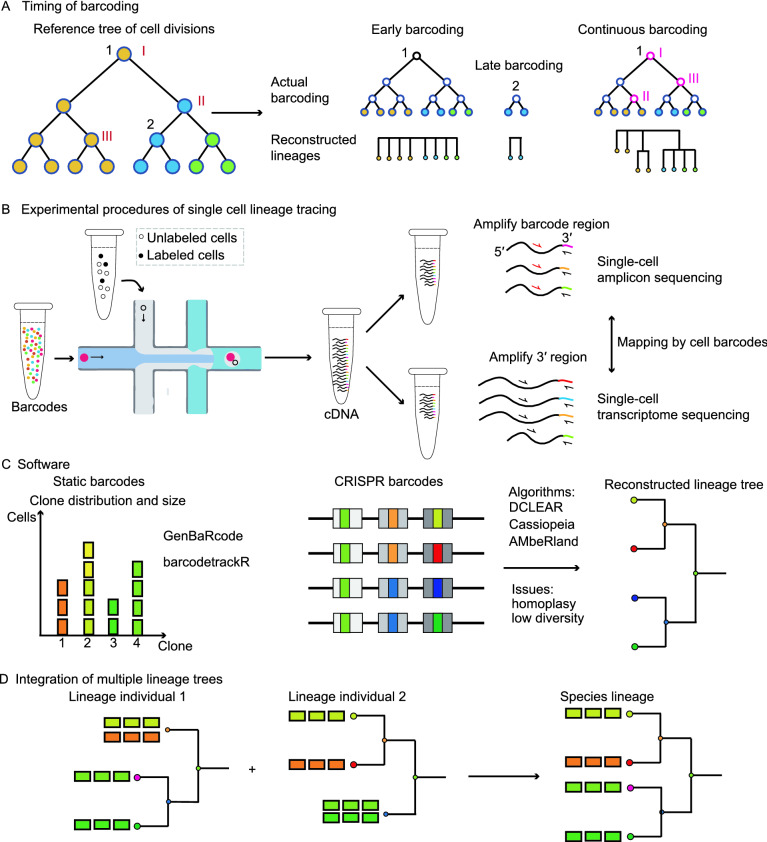
Technical considerations for applying single-cell lineage tracing technology. (A) The timing of barcoding affects the correlation granularity of cell types, which is determined by the internal nodes. Of note, the illustrated continuous barcoding does not recover all the subclonal information within a clone, as it’s difficult to reconstruct a complete cell phylogenetic tree. Different colors represent different cell types. (B) Common experimental practice for single-cell lineage tracing. Cells are captured and labeled with cell barcodes, then cDNAs are extracted and split into two aliquots, one aliquot for querying lineage relationships and one aliquot for interrogating cell types. (C) Some algorithms dealing with barcode analysis. (D) Multiple tree integration strategies based on cell types

The timing to initiate barcoding will have great influences on the resolution and accuracy of clonal reconstruction. According to the timing of barcoding in the concerned developmental process, there are four major modes of barcoding: early barcoding, intermediate barcoding, late barcoding, and continuous barcoding (Wagner and Klein, 2020). Early barcoding, which usually labels a pool of progenitors at the start of lineage tracing, facilitates the lineage tracing of specific clones but has difficulties in inferring the mitotic hierarchies between different cell types within a clone. Late barcoding has little contribution to understanding the internal structure of lineage trees, as it labels the end stage of development. Although intermediate barcoding could distinguish different cell types at late stages, the ideal continuous barcoding from early to late stages could help to build a multi-layer cell phylogenetic tree with lots of internal nodes, which record the valuable information of cell division history (Fig. 3A).

In practice, different kinds of barcodes are suited for different modes of barcoding according to their features. Generally, static barcodes have a large barcoding space, which could simultaneously label hundreds to thousands of clones through early barcoding. In contrast, as cumulative barcodes have informative sites to record the history of cell divisions, they are useful for obtaining cell phylogenetic trees through continuous barcoding. Based on the above considerations, integration barcodes and polylox barcodes are usually used to label a pool of heterogeneous progenitors *in vitro* and *in vivo*, respectively. Albeit CRISPR barcodes can be applied to label a stem cell population, they are mainly used for recording the different generations of descendants from a single ancestor (Fig. 2A). For the latter case, the timing of barcoding should be fine-tuned by adjusting the availability of the three components of the CRISPR/Cas9 system. This is because whether the CRISPR barcoding process captures the cell divisions depends on many factors, which include but are not limited to the number of informative sites, gene editing rates, and endogenous cell division rates (Salvador-Martinez et al., 2019). The direct way is to moderate the Cas9 activity by dox induction or heat shock induction, but *a priori* knowledge is required to select the best timing of barcoding (Bowling et al., 2020). Confining the Cas9 activity in a specific phase of the cell cycle such as the S/G_2_ phase should slow down the target exhaustion, as well as eliminate the post-mitotic editing events that may confound the lineage inference (Garcia-Marques et al., 2020). Mismatches between gRNAs and targets can also attenuate the gene-editing rates, which has been applied to depict animal embryogenesis across a longer time window (Chan et al., 2019; Salvador-Martinez et al., 2019). Homing gRNAs provide another means to increase the barcoding interval, though further improvement is needed to detect the homing RNA for barcode recovery (Kalhor et al., 2018; Kalhor and Church, 2019). A new design called gRNA cascade might offer a better solution by controlling the ordered activation of gRNAs, which is mediated by the single-strand anneal repair pathway after DNA double-strand breaks. By genetic switches to activate sequential gRNAs, this strategy slows down the exhaustion of targets (Garcia-Marques et al., 2019, 2020; Clarke et al., 2020). However, it’s still challenging to apply this strategy at present due to the unstable generation of new gRNAs through the single-strand anneal repair mechanism. Finally, distinct CRISPR barcodes are generated through cell divisions, so it’s impossible to capture the differentiation route of postmitotic cells. To maximize the CRISPR’s ability to dissect lineage hierarchies, one suggestion is to modulate the cell barcoding process in the stages of cell differentiation. In contrast to CRISPR barcodes, continuous barcoding by base editors suffers from low editing rates. As a result, modification of deaminase is usually required to increase the editing rates (Liu et al., 2021).

### Recovery rates of cells and barcodes

As SCLT requires the retrieval of both barcodes and RNA profiling, ensuring the simultaneous capture of cells and barcodes is quite essential for unleashing the power of SCLT (Fig. 3B). The current throughput of commercial scRNA-seq platforms (e.g., 10x Genomics) is around 10,000 cells per experiment, but high cost makes it impractical to sequence millions of cells, which may be necessary for organism-level tracing analysis. Insufficient sampling results in the doubt of whether selected cells can represent the whole population, thus affecting the accuracy and completeness of lineage couplings. Recently, two high throughput scRNA-seq techniques called SPLiT-seq and sci-RNA-seq have enabled the sequencing of millions of cells by labeling them using combinatorial indexing. However, further refinements of their gene detection rates are necessary for accurate downstream analysis (Rosenberg et al., 2018; Cao et al., 2019). Besides insufficient sampling, clonal tree reconstructions of CRISPR barcodes might also suffer from biased sampling, as current algorithms require inferring the shared InDels from a variety of barcodes. Finally, invasive barcoding strategies may have latent effects to the behaviors of barcoded cells, which is often difficult to evaluate. For example, CRISPR/Cas9-mediated gene editing could cause DNA double-strand breaks, leading to subsequent p53 activation and even cell death (Haapaniemi et al., 2018; Ihry et al., 2018; Chan et al., 2019), which might alter the lineage routes as well as lose the barcoded cells. To circumvent this problem, one compromised but potential solution is to use base editors, which create fewer allele states without DNA double-strand breaks (Eid et al., 2018; Jones et al., 2020).

A few factors also influence the retrieval of barcodes. As barcodes are usually amplified from cDNAs (Fig. 1A), the transcription efficiency of the barcodes is a critical point for the recovery of barcodes. Transgene silencing is a common issue for random transgenic insertions, and adding a transcription enhancer such as ubiquitous chromatin opening element before a constitutive promoter may attenuate this effect (Garrison et al., 2007; Chan et al., 2019). Targeted insertion at the *Rosa26* locus can also ensure the barcode expression. As for CRISPR barcodes, some minor factors for barcode loss may occur in PCR amplification. For example, large deletion removes the primer sequence, large insertion overwhelms the sequencing length (Egli et al., 2018). Another main concern is that a proportion of cells may be free of gene editing at the time of sequencing, leading to the loss of lineage information from these cells (Chan et al., 2019).

### Current computational methods

In SCLT experiments, clonal examination and single-cell transcriptome exploration are separately performed and then integrated by shared cell identifiers. As single-cell transcriptomic analysis is mature and has been reviewed extensively elsewhere (Wu and Zhang, 2020; Andrews et al., 2021), here we focus on computational methods of clonal analysis. Dependent on barcode types, different computational algorithms have been compiled to deal with clonal analysis (Fig. 3C). For experiments using integration barcodes, most of them build custom pipelines to analyze specific questions. Integration barcodes are similar to the UMIs (unique molecular identifiers) used in single-cell analysis. UMI-related software such as UMI-tools may offer a good start to evaluate the integration barcodes (Smith et al., 2017). Many other developed algorithms could also be tested. Specifically, Kong et al. built an analytical pipeline to present lineage hierarchies of limited layers for their barcoding technique called CellTagging, which employed several rounds of lentivirus infections to achieve sequential barcoding (Kong et al., 2020). However, in-house scripts are unfriendly for comparing results between different scenarios. To standardize the analytical procedure, two R-based programs were developed. genBaRcode focuses on basic error-correction and routine visualization of barcodes, while barcodetrackR steps further to interrogate the different aspects of clonal tracing by offering convenient functions to show pairwise lineage relationship, illustrate longitudinal clonal dynamics and infer lineage bias (Thielecke et al., 2020; Adair and Enstrom, 2021; Espinoza et al., 2021). As for polylox barcoding, an in-house algorithm was created to extract unique barcodes from long reads generated by third-generation sequencing (Pei et al., 2019).

The bioinformatics tools for CRISPR barcodes focus on tree reconstruction. Some investigations follow the common methods of phylogenetic tree reconstruction such as distance-based clustering, maximum parsimony, and maximum likelihood (McKenna et al., 2016; Kalhor et al., 2018; Feng et al., 2021), while others build custom algorithms with specific assumptions tailored to their barcode designs (Spanjaard et al., 2018; Bowling et al., 2020). Generally, distance-based methods aim to cluster cells to create lineage trees by distance matrix, which records the lineage information as measured by barcode dissimilarity. Alternatively, maximum-parsimony-based methods search for the tree topology with minimal total steps to generate observed InDel patterns. Besides tree topology, maximum-likelihood-based methods also attempt to infer branch length and mutation parameters. Although the above methods have been applied in different experimental settings, a few concerns remain. One major issue is the low diversity of CRISPR barcodes, which limits the tractable cells and is prone to result in barcode homoplasy. Another problem is sequence dropouts generated by large deletions across multiple targets (Fig. 1D), which reduce the accuracy of reconstructed trees by eliminating the previously recorded information (Jones et al., 2020; Gong et al., 2021). Recently, using both ground-truth datasets from a synthetic image-readable lineage recording technology and *in silico* datasets from simulated recording *C*. *elegans* or mouse development, Gong et al. reported systematic evaluations of dozens of methods which were roughly classified into three different groups (i.e., distance-based, maximum-parsimony-based, and machine-learning-based), setting a standard for the evaluation of future methods. As a new paradigm to reconstruct trees, machine-learning-based approaches leverage the features/mutation information in the training set to predict cell relationships. In this report, the authors summarized that some of the best-performing methods were distance-based DCLEAR, maximum-parsimony-based Cassiopeia, and machine-learning-based AMbeRland (Gong et al., 2021). Notably, Cassiopeia has already been demonstrated to be a powerful tool for building cell lineage trees in mouse embryogenesis (Chan et al., 2019; Jones et al., 2020). In terms of base-editing barcodes generated by SMALT design, the maximum-likelihood method was used to reconstruct the cell phylogenetic tree (Liu et al., 2021).

### Integration of multiple lineage trees

A thorough and ambitious understanding of cell lineage is to illustrate the relationship of every single cell in an organism in a single lineage tracing experiment, which is impractical for current technology. A more practical way is to integrate information from multiple lineage tracing experiments, as the current knowledge of developmental lineages is also assembled from multiple lineage experiments with various techniques. Lower animals such as *C*. *elegans* have an invariant cell lineage tree, thus assembling a lineage tree from multiple small lineage trees is feasible (Sulston et al., 1983). However, apart from technical limitations, the cell lineage of human development isn’t invariant *per se* due to the stochasticity/plasticity of each cell fate decision (Zechner et al., 2020), making it unrealistic to assemble multiple small lineage trees collectively to create a complete lineage tree in the organismal level. In this regard, cell-type-based lineage trees might be appropriate for most tracing cases (Fig. 3D). In this kind of lineage tree, the leaves should be cell populations of specific cell types instead of a single cell (Wagner and Klein, 2020). Although currently focusing on mutual calibrations of lineage trees or cell trajectories, some pioneer efforts have shed light on the integrative datasets to build a cell-type based lineage tree by extracting the information from both lineages and transcriptomes. For example, a statistical framework, LinTiMaT, attempted to resolve lineage ambiguities when integrating different individual lineage trees into a single invariant tree by incorporating gene expression information across different experiments (Zafar et al., 2020; Forrow and Schiebinger, 2021).

## Summary and perspectives

SCLT integrates the information of both clonal relationships and single-cell transcriptomics, which greatly improves the resolution and accuracy compared to traditional lineage tracing. Its enormous throughput and content help to reveal the phylogenetic foundations of biological processes that encompass many cell generations at unprecedented resolutions and scales. For the experimentally tractable system, three major types of barcodes and their combinations have been exploited in querying the molecular mechanisms of cell fate determination. Among the discussed barcoding strategies, CRISPR barcodes and base-editing barcodes are uprising for their high information capacity and cumulative features, despite more studies are necessary to achieve abundant barcode diversity, easy detection, high precision, and long-term barcoding capacity (Espinosa-Medina et al., 2019; Liu et al., 2021). As a proof-of-concept, current SCLT experiments focus on embryogenesis and hematopoiesis, future interrogations should witness its broad application in animal development, regeneration, tumorigenesis, and stem cell dynamics. However, obtaining an accurate and systematic lineage tree of a species remains a challenging task for current practices of SCLT, and new designs are necessary for further improvements.

**Figure. 4:**
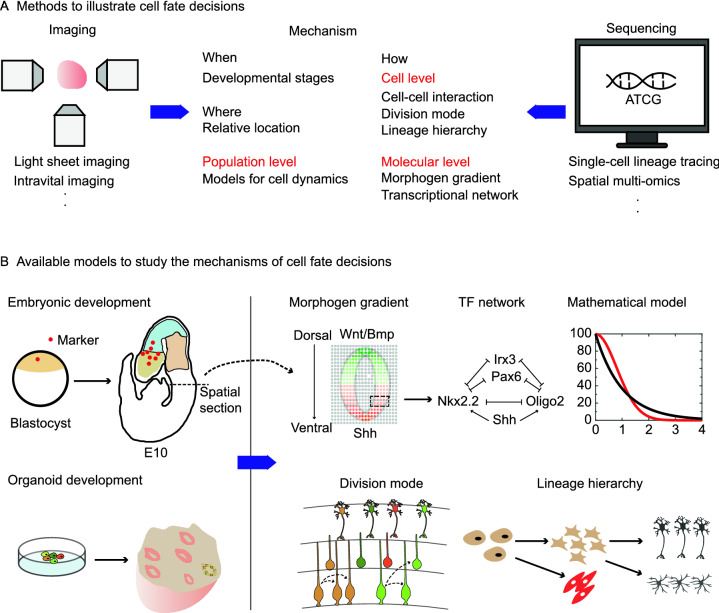
Integrating multiple technologies to illustrate developmental mechanisms. (A) Available imaging and sequencing methods. Imaging methods are convenient to track developmental events across different stages and record the spatial locations of cells, while sequencing methods can interrogate molecular profiles of cells in a high throughput manner. (B) Organoid model is complementary with *in vivo* model to study developmental mechanisms in multiple aspects [TF (transcription factor) network is adapted from (Sagner and Briscoe, 2019)]

Recently, the fast development of spatial transcriptomics has shed light on the understanding of the spatial organization of cell types and their lineage relationships (Peng et al., 2019; Peng et al., 2020; Marx, 2021). Several pioneering investigations have proven the concept of combining lineage tracing and spatial transcriptomics by imaging-based readouts (Chow et al., 2021; Frieda et al., 2017). A recent study integrating lineage tracing and spatial transcriptomics has demonstrated that the closely located cells tend to share lineage origins in cerebral organoids (He et al., 2022). This phenomenon may not be shared by the development of neural crest cells and some other long-traveling cells, as their derivatives are spread around the whole organism instead of compact clusters. Nevertheless, the complicated developmental processes may be illustrated by the microscope-based real-time lineage tracing, as this technology can offer valuable information about the cell division number, cell division rate, cell location, cell history, and so on (Denoth-Lippuner et al., 2021; Huang et al., 2021). Anchored by cell’s location, combining lineage-information-based spatial transcriptomics and *in toto* imaging will build a connection between ground-truth cellular history and final molecular interrogation. Future advances to integrate SCLT, spatial multi-omics, and imaging techniques should boost the synthesized studies of animal development and disease modeling in both space and time (Fig. 4A).

In the last few years, the scientific community has witnessed the fast development of technologies utilizing organoids. As human stem-cell-derived organoids could mimic the organ/tissue development of humans, SCLT on human organoids may pave the path to study the lineages of human cell types, which are usually extracted from the incomplete somatic mutations nowadays (Fig. 4B). More importantly, SCLT on human organoids displays the following advantages. First, specific organoids might describe the lineage transition of human cell types more accurately and controllable than their mouse *in vivo* equivalents, especially for those complex organs such as the human brain (Lancaster et al., 2013). Second, manual modulations of the culture condition enable studying different phases of organ development step by step. For example, current breakthroughs allow for studying the cardiogenesis at multiple overlapping stages such as on gastruloids, foregut-heart organoids, or heart organoids (Drakhlis et al., 2021; Rossi et al., 2021; Hofbauer et al., 2021a, 2021b). As each organoid system contains several consecutive steps of cardiac development, it’s possible to illustrate the lineage path and molecular transitions of heart differentiation by sticking their lineages together. Third, since organoids are cultured in a dish, it’s convenient to combine cutting-edge imaging techniques to track cell behaviors. In a word, complementary studies of embryonic development and organoid development would show a clearer picture of cell fate decisions in the future.

## Declarations

This work was supported in part by National Key R&D Program of China (2018YFA0801402, 2018YFA0107200), the "Strategic Priority Research Program" of the Chinese Academy of Sciences (XDA16020404), Guangdong Basic and Applied Basic Research Foundation (2019B151502054), Frontier Research Program of Bioland Laboratory (Guangzhou Regenerative Medicine and Health Guangdong Laboratory, 2018GZR110105013), Jiazi Research Innovative Project of Bioland Laboratory (2019GZR110108001), Science and Technology Planning Project of Guangdong Province (2020B1212060052).

### Abbreviations

CNV, copy number variation; CRISPR, clustered regularly interspaced short palindromic repeats; CRISPR BC, CRISPR barcode; FACS, fluorescence-activated cell sorting; FP, fluorescent protein; HSCs, hematopoietic stem cells; InDel, insertion and deletion; IntBC, integration barcode; MF, mosaic fractions; pA, polyA tail; polyloxBC, polylox barcode; SCLT, single-cell lineage tracing; scRNA-seq: single-cell RNA sequencing; SNP, single nucleotide polymorphism; SNVs, single nucleotide variations; TF, transcription factor; UGI, uracil DNA glycosylase inhibitor; UMAP, uniform manifold approximation and projection; UMIs, unique molecular identifiers; UTR, untranslated region.
